# Pathological complete response in esophageal squamous cell carcinoma complicated by malignant fistula via neoadjuvant immunotherapy, chemoradiotherapy, and nutritional support: a case report

**DOI:** 10.3389/fimmu.2026.1750544

**Published:** 2026-02-11

**Authors:** Nanxi Li, Mei Lan, Wenxiu Yao, Yixiang Zhu, Qifeng Wang

**Affiliations:** 1 Department of Radiation Oncology, Sichuan Clinical Research Center for Cancer, Sichuan Cancer Hospital & Institute, Sichuan Cancer Center, School of Medicine, University of Electronic Science and Technology of China, Chengdu, Sichuan, China; 2 Department of Thoracic Medical Oncology, Sichuan Clinical Research Center for Cancer, Sichuan Cancer Hospital & Institute, Sichuan Cancer Center, School of Medicine, University of Electronic Science and Technology of China, Chengdu, Sichuan, China

**Keywords:** case report, esophageal fistula, esophageal squamous cell carcinoma, neoadjuvant immunotherapy and chemoradiotherapy, pathological complete response

## Abstract

Esophageal cancer presents a formidable global health challenge, particularly when complicated by esophageal fistulas, which historically confer a dismal prognosis and severely limit therapeutic options. We report a challenging case of locally advanced esophageal squamous cell carcinoma (ESCC) complicated by an esophageal fistula that ultimately achieved a pathological complete response (pCR). The patient, a male in his 50s, presented with cough and dysphagia and was diagnosed with Stage IVA ESCC. Notably, an esophageal fistula was identified at the initial presentation, posing a threat to treatment. Aggressive enteral nutritional support via nasogastric tube placement and subsequent percutaneous endoscopic gastrostomy (PEG) was promptly initiated. This allowed the patient to successfully complete neoadjuvant therapy consisting of the anti-PD-1 antibody tislelizumab, paclitaxel, carboplatin, and concurrent intensity-modulated radiation therapy (IMRT). Following this multimodal regimen, the patient was reassessed as resectable following response to therapy status. Subsequent radical esophagectomy revealed no residual tumor cells in the primary lesion or dissected lymph nodes (ypT0N0M0), confirming pCR. Immunohistochemical analysis of pre-treatment biopsies demonstrated PD-L1 positivity and high infiltration of CD8+ T cells, suggesting that a robust immune-active microenvironment favored the efficacy of PD-1 blockade. This case underscores the feasibility of integrating immunotherapy with chemoradiotherapy in ESCC patients complicated by esophageal fistulas when supported by rigorous nutritional management.

## Introduction

Esophageal cancer is a major public health issue globally, with high incidence and mortality rates. The 2024 National Cancer Report indicates that China reported about 4.82 million new cancer cases and 2.57 million cancer-related deaths in 2022, with 224,000 cases of esophageal cancer, leading to approximately 187,500 deaths (1). The esophagus’s structural characteristics make it prone to anastomotic ischemia, increasing fistula risk, which ranges from 4.3% to 8.1%. Without aggressive treatment, the median survival time of patients with esophageal fistula will be shortened. For these patients, effective treatment options are still lacking. We present a case of a patient with esophageal cancer diagnosed with an esophageal fistula. After nutritional support, the patient successfully completed two cycles of induction chemoimmunotherapy, following by radiotherapy and chemoimmunotherapy, ultimately undergoing surgery and achieving pathological complete response (pCR).

## Case presentation

### Patient information

A 50-year-old male presented in 2024 with dysphagia of unknown cause. While the symptom initially manifested as an obstruction sensation during rapid eating, it gradually progressed to difficulty with solid foods. He denied B symptoms such as fever, night sweats, or weight loss. Although he had no nausea or vomiting, he suffered from severe coughing with dark red blood-tinged sputum and experienced intermittent abdominal discomfort. Physical examination was unremarkable. The patient had a history of smoking (20 cigarettes/day for over 20 years, ceased 5 years prior) and occasional alcohol consumption. His past medical history included surgery for a left ankle fracture and perianal abscess resection six years ago. He denied any history of hypertension, diabetes, coronary heart disease, tuberculosis, or hepatitis. The Karnofsky Performance Status (KPS) score at admission was 80.

### Timeline

The clinical course and treatment timeline of the patient are summarized in **Table 1**.

**Table 1:** Timeline table.

Event	Date	Details
Symptom onset	2024	Dysphagia (worsened with rapid eating), coughing with dark red blood-tinged sputum, intermittent abdominal discomfort
Diagnosis	June 12, 2024	Esophageal squamous cell carcinoma (clinical T4N2M0, Stage IVA) via CT, endoscopy, and biopsy
1st cycle of chemoimmunotherapy	June 20, 2024	Paclitaxel 135 mg/m² d1+ Carboplatin at an AUC of 4 d1+ Tirelizumab 200 mg d1
2nd cycle of chemoimmunotherapy	July 12, 2024	Paclitaxel 135 mg/m² d1+ Carboplatin at an AUC of 4 d1+ Tirelizumab 200 mg d1
Detection of esophageal fistula	August 2, 2024	CT showed incomplete continuity of the right lateral esophageal wall; endoscopy revealed localized depression (36 cm from incisors) with bubble overflow
Percutaneous endoscopic gastrostomy (PEG) placement	August 6, 2024	Performed due to nasogastric tube-related pharyngeal discomfort and need for long-term nutritional support
3rd cycle of chemoimmunotherapy	August 14, 2024	Paclitaxel 135 mg/m² d1+ Carboplatin at an AUC of 4 d1+ Tirelizumab 200 mg d1
Initiation of IMRT	August 16, 2024	Intensity-modulated radiation therapy targeting primary tumor and mediastinal lymph nodes
Completion of IMRT	September 20, 2024	Total dose: 1.8 Gy × 23 fractions (total 41.4 Gy)
4th cycle of chemoimmunotherapy	September 6, 2024	Paclitaxel 135 mg/m² d1+ Carboplatin at an AUC of 4 d1+ Tirelizumab 200 mg d1
Radical surgery	October 10, 2024	Three-incision radical esophagectomy + thoracic duct ligation + esophageal reconstruction + right lower lung lobe wedge resection
Pathological result	Post-October 10, 2024	ypT0N0M0, pathological complete response (pCR); no residual cancer in primary site, lymph nodes, or resection margins
1st postoperative follow-up (CT)	November 19, 2024	Postoperative changes, mild mediastinal fluid accumulation, reduced left pleural effusion, no recurrence
One year postoperative follow-up (CT)	2025 (5 months after November 2024)	No tumor recurrence, improved pulmonary inflammation; KPS score 90

### Clinical findings and diagnostic assessment

**Figure 1:**
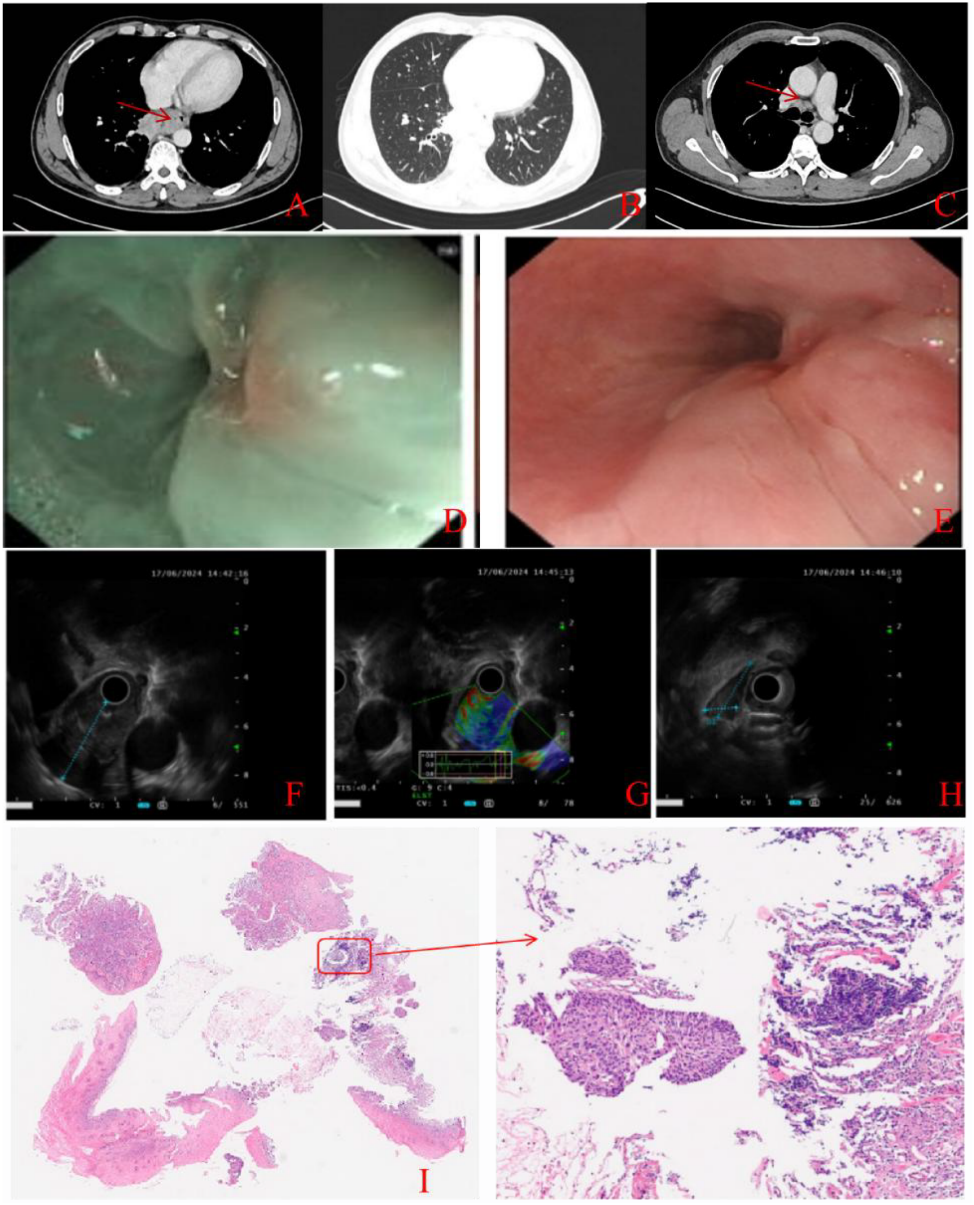
Imaging examination before patient treatment. **(A-C)** CT image on June 12, 2024, **(A)** shows the primary lesion (arrow indicates the primary tumor), **(B)** showing the lung window and **(C)** showing the mediastinal lymph nodes (arrow indicates the mediastinal lymph nodes, station 4R). **(D, E)** Upper gastrointestinal endoscopy. **(F-H)** Ultrasound gastroscopy images at diagnosis, with **(F)** showing the Ultrasound of lesion, **(G)** showing the Elastrography. **(H)** showing the Mediastinal ultrasound. **(I)** Pathological image at diagnosis.

On June 12, 2024, computed tomography (CT) scans of the upper chest and abdomen showed significant thickening of the esophageal wall in the lower thorax, with possible invasion into the right posterior mediastinum and adjacent lower lobe of the right lung. Multiple lymphadenopathies in the mediastinum, right hilar region, and hepatogastric space suggested metastasis (**Figures 1A–C**). Upper gastrointestinal endoscopy revealed an extrinsic bulge on the posterior wall, 34–38 cm from the incisors, with an irregular mucosal surface (**Figures 1D, E**). The lesion’s nature is uncertain, though chronic non-atrophic gastritis was noted. Ultrasound gastroscopy demonstrated marked esophageal wall thickening at 34–38 cm, with the thickest portion measuring about 35.6 mm, indicating invasion into the extra-adventitia. Scattered lymph nodes around the lesion had a maximum diameter of 12.5 mm (**Figures 1F–H**). Biopsy confirmed squamous cell carcinoma, with immunohistochemical analysis showing P40 (+), P53 (+, 100%), P16 (-), P63 (+), CK5/6 (+), CK (+), and KI-67 (+, 30%) (**Figure 1I**). Following departmental discussions, the patient was diagnosed with squamous cell carcinoma of the lower thoracic esophagus, with suspected lung invasion, mediastinum and hepatogastric space lymph node metastasis (clinical T4N2M0, Stage IVA).

### Therapeutic intervention

**Figure 2:**
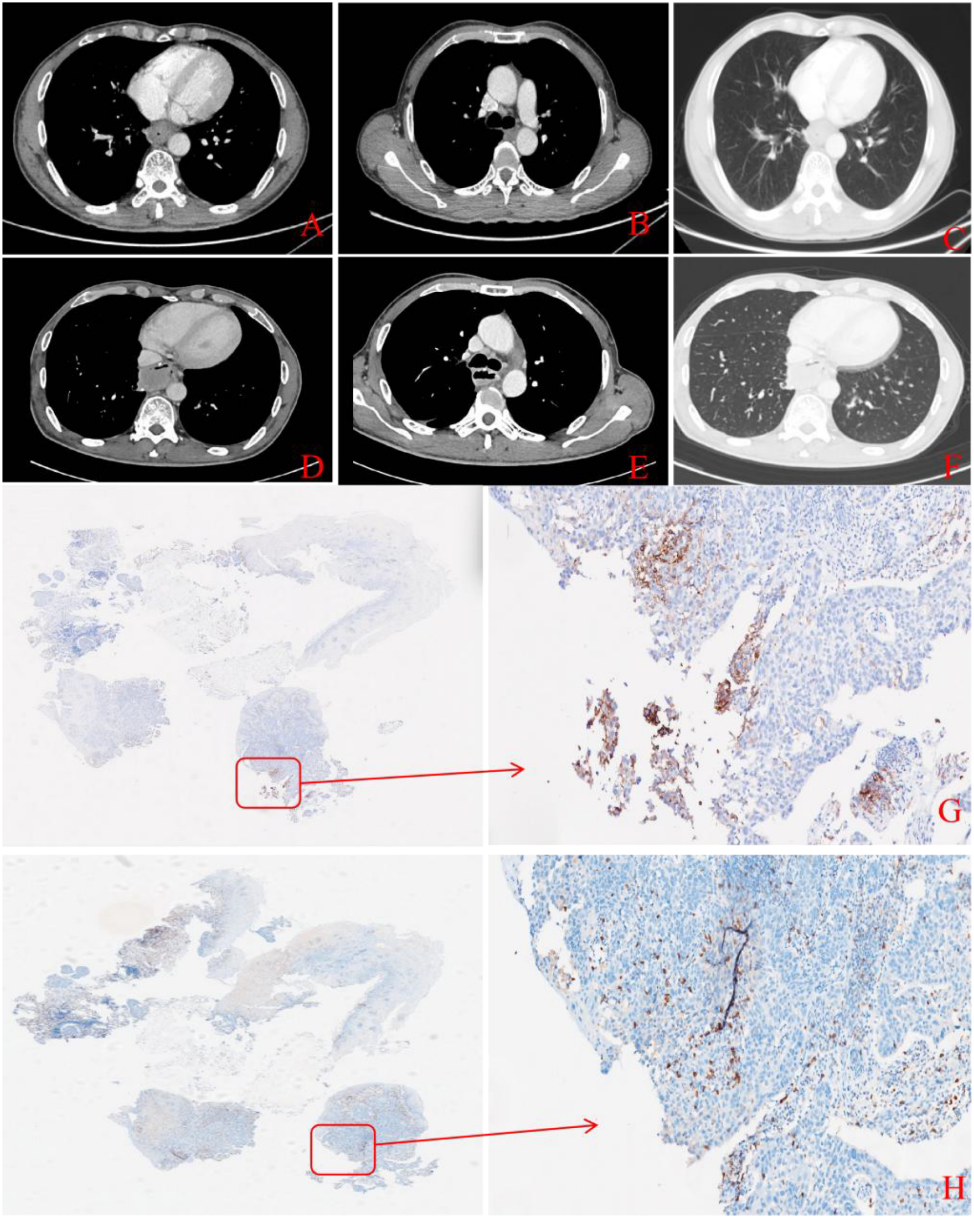
Imaging examination after treatment and immunohistochemical staining image before treatment. **(A-C)** are preoperative CT images on October 8, 2024, with **(A)** showing the primary lesion **(B)** showing the mediastinal lymph nodes and **(C)** showing the lung window. **(D-F)** are CT images after surgery, **(D)** shows the location of the original lesion before, **(E)** shows the previous location of the mediastinal lymph nodes and **(F)** showing the lung window. **(G)** Image of the patient with PD-L1 staining of pre-treatment biopsy specimens. (magnification, ×10) **(H)** Image of the patient with CD8 staining of pre-treatment biopsy specimens. (magnification, ×10).

Considering that the patient has an esophageal fistula, a nasogastric tube was inserted. With the implementation of optimal supportive measures, including nutritional support and anti-infective therapy, the patient’s overall health status demonstrated marked improvement. Given the advanced disease stage, the focus shifted to systemic therapy. The patient underwent the first and second cycles of chemoimmunotherapy on June 20, 2024, and July 12, 2024, respectively. The treatment regimen included paclitaxel 135 mg/m² d1, carboplatin at an AUC of 4 d1, and tislelizumab (an programmed death receptor-1 [PD-1] blockade) 200 mg d1, once every three weeks. A follow-up CT scan on August 2, 2024 showed uneven thickening of the lower thoracic esophagus wall, with possible invasion into the right posterior mediastinum and lower lobe of the right lung. The local right lateral wall was incompletely continuous, suggesting potential fistula formation. Although the tumor size decreased compared to the previous scan, an esophageal fistula had developed. A re-examination of the upper gastrointestinal endoscopy revealed a localized depression at 36 cm from the incisors and evidence of bubble overflow. Due to pressure from the nasogastric tube on the pharyngeal mucous membrane, the patient’s coughing and sore throat symptoms worsened. Given the upcoming chemoradiotherapy and the need for adequate nutrition while managing pain, a gastrostomy was deemed necessary. An endoscopic percutaneous gastrostomy was performed on August 6, 2024. The patient then completed the third and fourth cycles of chemoimmunotherapy on August 14 and September 6, 2024, respectively, with a regimen of paclitaxel 135 mg/m² d1, carboplatin at an AUC of 4 d1, and tislelizumab 200 mg d1. The patient failed to present at the hospital on the scheduled date, resulting in a longer-than-usual interval between the second and third cycles of chemotherapy. Concurrently, from August 16 to September 20, 2024, the patient received intensity-modulated radiation therapy (IMRT) targeting the primary esophageal tumor and mediastinal lymph nodes, delivering a dose of 1.8 Gy over 23 fractions. A follow-up CT scan conducted on October 8, 2024, showed uneven thickening of the lower thoracic esophageal wall, reduced in size from previous scans. Enlarged lymph nodes in the mediastinum, right hilar region, hepatogastric ligament, and retroperitoneum were noted, with some showing size reduction (**Figures 2A–C**). After multidisciplinary discussion, the patient is considered for potential surgical treatment.

On October 10, 2024, the patient underwent radical esophagectomy with three-incision approach (neck, thorax, and abdomen), along with thoracic duct ligation, esophageal reconstruction, and wedge resection of the right lower lung lobe. Postoperative pathological: 1. Lymph node summary: Total lymph nodes examined: 22; Total positive lymph nodes: 0 (all 22 nodes were negative); Station-wise breakdown: Right recurrent laryngeal nerve LN: 0/1; Station 8 LN: 0/1; Station 7 LN: 0/2; Station 15 LN: 0/1; Left recurrent laryngeal nerve LN: 0/1; Periesophageal LN: 0/2; Perigastric LN: 0/2; Station 16 LN: 0/4; Station 17 LN: 0/9; 2. Primary tumor bed and regression: The primary esophageal tumor bed showed fibrous tissue proliferation with chronic inflammatory cell infiltration, consistent with a complete pathological response (ypT0) following neoadjuvant therapy; no residual tumor cells were identified. All surgical margins (esophageal, gastric, and radial margins) were negative for carcinoma. No lymphovascular invasion or perineural invasion was detected. 3. Upper esophageal stump and right lower lung wedge resection findings: Upper esophageal stump: A spindle cell tumor was identified, confirmed as a leiomyoma by immunohistochemistry (stains: SMA (+), Desmin (+), S100 (-), CD34 (-), CD117 (-), Dog-1 (-), SDHB (+), Ki-67 (~1%)). This is a benign lesion unrelated to the esophageal squamous cell carcinoma. Right lower lung wedge resection: No tumor metastasis or neoplastic lesions were present; only inflammatory changes were noted. Overall, the pathology suggests ypT0N0M0 stage, indicating pCR.

### Follow-up and outcomes

A follow-up CT scan on November 19, 2024, showed postoperative changes in the esophagus, with slightly thickened anastomotic soft tissue and blurred surrounding fat planes. There was a slight accumulation of fluid in the mediastinum, consistent with prior findings. Changes were also noted in the lower lobe of the right lung, along with a trace of pleural effusion on the left side, which had significantly decreased compared to earlier scans (**Figures 2D–F**). One year later, during a follow-up visit, the CT scan showed no signs of tumor recurrence, and pulmonary inflammation had improved. The patient’s postoperative recovery was satisfactory. Throughout treatment, the primary adverse events included grade 1 nausea, grade 1 vomiting, and grade 3 leukopenia and neutropenia (CTCAE v5.0). The nadir occurred on September 14, 2024, with a white blood cell count of 1.1 × 10^9^/L and an absolute neutrophil count of 0.6 × 10^9^/L. All toxicities resolved following symptomatic treatment. No other toxic side effects were reported, indicating the treatment’s safety. Currently, the patient’s condition is stable, with occasional shortness of breath and cough. The patient reports no weight loss, with a KPS score of 90 and a good appetite.

## Discussion

Currently, chemoradiotherapy has become the standard treatment for locally advanced esophageal squamous cell carcinoma (ESCC). However, it is difficult for patients with esophageal cancer complicated by fistula to complete these treatments smoothly. Previous study indicates that the median overall survival (OS) for patients with esophageal fistula is approximately 6 to 8 months, significantly shorter than the 26.8 months observed in patients without fistula (2). We present a case of an esophageal cancer patient with a fistula. Supported by enteral nutrition, the patient underwent and tolerated neoadjuvant treatment comprising chemotherapy, immunotherapy, and radiotherapy. Subsequent surgery confirmed a pCR. This favorable result demonstrates the efficacy of a multidisciplinary approach and tailored therapeutic strategy.

Standard management approaches for esophageal fistula involve surgical excision, stent insertion, gastrostomy, nasogastric tube utilization, and supportive care (3). Xu et al. (4) indicated that post-fistula treatments—including radiotherapy (HR 0.40, 95% CI 0.20–0.80), chemotherapy (HR 0.24, 95% CI 0.08–0.72), or combined chemoradiotherapy (HR 0.10, 95% CI 0.05–0.19)—were associated with significantly improved survival compared to no such interventions. Nevertheless, definitive clinical guidelines are absent regarding whether chemotherapy or radiotherapy post-fistula genuinely enhances patient outcomes, and it remains unclear which treatment modality is preferable.

CROSS study (5) confirms neoadjuvant chemoradiotherapy can improve the pCR rate and OS for patients with locally advanced esophageal cancer. The TD - NICE study (6)confirmed the safety and efficacy of tislelizumab combined with chemotherapy in neoadjuvant therapy for resectable ESCC, with a high postoperative major pathological response (MPR) rate. The NICE1 study (7) also compared neoadjuvant chemoimmunotherapy and concurrent chemoradiotherapy, and the results showed that the two-year overall survival rate of patients with chemoimmunotherapy was 81.3%, compared with 71.3% of chemoradiotherapy, and the disease-free survival rate for both was 73.9% vs 63.4%, respectively, indicating the clear advantages of immune therapy. A recent study to explore the safety and efficacy of toripalimab plus chemoradiotherapy for patients with stage IV ESCC. The results indicated that the median progression-free survival (PFS) was 9.8 months and the OS was not reached (8). The combination treatments did not significantly increase the risk of esophageal fistula. In this case, initial nutritional support was provided, followed by two cycles of induction chemoimmunotherapy. Compared to immediate initiation of chemoradiation, starting with symptomatic nutritional support and chemoimmunotherapy may allow time for fistula healing. This is then followed by chemoradiation and immunotherapy, ultimately leading to surgery and achievement of pCR.

At the same time, we investigated the reasons why this patient responded well to the treatment. We conducted programmed cell death 1 ligand 1 (PD-L1) (**Figure 2G**) and CD8 (**Figure 2H**) immunohistochemical staining on the patient’s pre-treatment biopsy specimens. The results revealed PD-L1 positive and highly CD8+T cells infiltration. Song et al. demonstrated that chemoimmunotherapy significantly improved objective response rate, PFS and OS in patients with PD-L1-positive advanced ESCC (9). Li et al. (10) revealed that CD8 Tex-SPRY1 cells presenting precursor depletion can enhance the anti-tumor immune response and are an effective predictive biomarker for PD-1 blockade-based neoadjuvant immunotherapy. Before neoadjuvant PD-1 blockade treatment, the level of CD8 Tex-SPRY1 cell infiltration in patients in the complete remission group was significantly higher than that in the non-complete remission group. Additionally, high CD8+T cells infiltration reflects a strong anti-tumor immune response, inhibiting tumor cell proliferation and improving patient outcomes. For this patient, the good efficacy may be attributed to the PD-L1 positive and highly CD8+T cells infiltration. We acknowledge that PD-L1 expression and CD8+T cells infiltration does not prove causality for pCR. This finding supports a hypothesis regarding the potential predictive value of these biomarkers, which requires validation in larger cohorts. Furthermore, it is difficult to distinguish the specific contribution of each therapeutic component (chemotherapy, immunotherapy, and radiotherapy) to the observed pCR. Given the complex interplay between these modalities, the complete response is likely the result of a synergistic effect rather than the action of a single agent. Additionally, surgery to remove the primary lesion and fistula may benefit for this patients. This case serves as a reference for individualized therapy in ESCC patients with esophageal fistula; however, further exploration in larger samples and prospective studies is necessary. To evaluate the safety of neoadjuvant chemoradiotherapy combined with immunotherapy in this specific patient population, we have launched a prospective, single-arm phase I clinical trial (SCCHEC-02-2025-128).

## Patient perspective

Receiving a diagnosis of esophageal cancer was overwhelming, and the development of an esophageal fistula made me fear that I would not be able to eat or survive the aggressive treatment. The constant coughing and dysphagia were physically and mentally draining. However, the medical team’s decision to place a nasogastric tube and perform a gastrostomy was a turning point. Although I was initially hesitant about it, it provided me with adequate nutritional support and allowed me to maintain my strength, thereby enabling me to tolerate the subsequent immunotherapy, chemotherapy and radiation. I felt fully supported throughout the immunotherapy cycles. Learning that the postoperative pathology showed no residual cancer cells was beyond my wildest expectations. I am now able to eat normally and have returned to my daily life, filled with gratitude for this personalized treatment plan.

## Methods

### PD-L1 assay

The PD-L1 immunohistochemistry (IHC) was performed using the 22C3 clone on the Dako Autostainer Link 48 platform. Tissue sections were deparaffinized and rehydrated through a graded alcohol series, followed by antigen retrieval in sodium citrate buffer. The sections were then blocked with 5% goat serum for 30 minutes at room temperature and incubated with primary antibodies overnight at 4°C. IHC staining was carried out using horseradish peroxidase (HRP)-conjugated secondary antibodies (anti-rabbit IgG, ZSGB-BIO, Cat# PV-6001; anti-mouse IgG, ZSGB-BIO, Cat# PV-6002) with DAB as the chromogen. Finally, the stained images were scanned and captured using Image Viewer (version 3.2) and analyzed with ImageJ software (National Institutes of Health, USA). The scoring criterion was based on the Combined Positive Score (CPS).

### CD8 quantification

CD8 was quantitatively analyzed using a digital quantification platform. The density was calculated as 71 cells/mm², which is significantly higher than that in the adjacent normal tissue (used as a control reference).

## Data Availability

The original contributions presented in the study are included in the article. Further inquiries can be directed to the corresponding author/s.
